# Novel Enzyme Replacement Therapies for Neuropathic Mucopolysaccharidoses

**DOI:** 10.3390/ijms21020400

**Published:** 2020-01-08

**Authors:** Yuji Sato, Torayuki Okuyama

**Affiliations:** 1 Research and Development, JCR Pharmaceuticals, Hyogo 659-0021, Japan; 2 Centre for Lysosomal Storage Diseases, National Centre for Child Health and Development, Tokyo 157-8535, Japan; okuyama-t@ncchd.go.jp

**Keywords:** neuropathic mucopolysaccharidosis, neurodegeneration, enzyme replacement therapy, blood brain barrier, transferrin receptor, insulin receptor, transcytosis

## Abstract

Although the advent of enzyme replacement therapy (ERT) for mucopolysaccharidoses (MPS) has paved the way for the treatment for these hereditary disorders, the blood brain barrier (BBB) has prevented patients with MPS involving the central nervous system (CNS) from benefitting from ERT. Therefore, finding ways to increase drug delivery into the brain across the BBB remains a crucial challenge for researchers and clinicians in the field. Attempts have been made to boost brain uptake of enzymes by targeting various receptors (e.g., insulin and transferrin), and several other administration routes have also been tested. This review summarizes the available information on clinical trials (completed, ongoing, and planned) of novel therapeutic agents with efficacy against CNS symptoms in neuropathic MPS and also discusses the common associated challenges and pitfalls, some of which may help elucidate the pathogenesis of the neurodegeneration leading to the manifold CNS symptoms. A summary of current knowledge pertaining to the neuropathological progression and resultant neuropsychiatric manifestations is also provided, because it should be useful to ERT researchers looking for better approaches to treating CNS lesions in MPS.

## 1. Introduction

The advent of enzyme replacement therapy (ERT) for lysosomal storage disorders, spearheaded in 1991 by the introduction of glucocerebrosidase for Gaucher disease and followed in 2003 by that of laronidase (α-iduronidase) for mucopolysaccharidoses (MPS) type I [1], has paved the way for long awaited pharmacotherapies for these hereditary disorders. These therapies compensate for genetic deficiency by preventing the accumulation of the substrates that inflict damage on the systemic structures and functions of patients. However, patients with MPS with central nervous system (CNS) involvement (also known as neuropathic MPS, i.e., MPS I, II, III, and VII) have been unable to benefit from ERT, because the blood brain barrier (BBB) prevents large molecules, including enzymes, from penetrating the brain parenchyma, which in turn prevents ERT from acting against substrate accumulation in the CNS. Therefore, elucidating the CNS pathology that leads to neurocognitive symptoms remains a significant challenge both in research and clinical practice [2].

Efforts have been made to boost brain uptake of drugs across the BBB by targeting various receptors (e.g., insulin and transferrin) located on the vascular endothelial cells where, by way of transcytosis, modified enzymes are delivered into and exert effects on the brain. Positive results have been reported in preclinical and clinical studies on MPS-I [3] and MPS-II [4,5]. Administration routes other than intravenous injection (e.g., intrathecal [6,7] and intracerebroventricular [8] injections) have also been attempted with the aim of delivering enzymes directly into the brain, but they invariably involve significant practical difficulties for both physicians and patients. Based on reports of completed, ongoing, and planned clinical trials, this paper summarizes recent advances in novel BBB penetrating ERTs to address neurodegeneration and CNS symptoms in neuropathic MPS.

Research carried out to develop novel therapeutics for neuropathic MPS can shed light on the nature of progressive neurodegeneration itself: although the basic etiology is clearly demarcated as a genetic deficiency in an enzyme that results in substrate accumulation, the progression of degeneration and the pathogenesis of the resultant manifold CNS symptoms are still in need of further elucidation, which is likely to come from research aimed at developing novel ERTs for neurodegeneration. For this reason, this paper also includes a brief summary of reported neuropathological findings and corresponding neuropsychiatric manifestations, with some suggestions on possible ways forward to better understand and address neuropathic MPS.

## 2. Current Treatments for Neuropathic MPS

### 2.1. General and Specific Treatments

Treatments for MPS are out of necessity multidisciplinary because of the multi-systemic nature of the disease. They can be divided into two major categories, general and specific [1,9]. General treatments involve supportive and palliative therapy for the various systemic and CNS symptoms associated with MPS. Before ERT became available, general treatments were used for all cases of MPS, but they are still used today for cases that cannot be treated with ERT and also for cases where symptoms persist despite ERT.

General treatments include symptomatic management of neuropsychiatric symptoms (e.g., sleep disturbance, seizures, mood disturbance, agitation, and aggression) with psychotropic agents, mood stabilizers, and anticonvulsants [10,11]. Surgical interventions are required for some systemic symptoms and physical disabilities (e.g., bone pain, ligamentous injury, abdominal hernias, hydrocephalus, and compression neuropathies), and patients with chronic physical disabilities and deformities often require physiotherapy to maintain physical function and the activities of daily living.

The specific treatments currently available for MPS, apart from ERT, include hematopoietic stem cell transplantation, substrate reduction therapy, chaperon therapy, and gene therapy. These are covered elsewhere in this Special Issue, so this paper focuses on an ERT for neuropathic MPS.

### 2.2. Current Enzyme Replacement Therapies and Their Limitations

The goal of ERT is to compensate for genetic enzyme deficiency by regularly administering recombinant enzymes, thereby preventing, improving, stabilizing, or decelerating the various systemic symptoms generated by accumulated substrates and the associated pathophysiology. At the time of writing, four recombinant enzymes have been approved for the treatment of MPS in most industrialized nations: laronidase for MPS I, idursulfase for MPS II, galsulfase for MPS VI, and vestronidase for MPS VII. No recombinant enzyme products have yet been developed to treat the three other types of MPS. For types I, II, III, and VII, even the currently available enzymes can only address the systemic symptoms, leaving neurodegeneration to progress unhindered. As detailed later, progressive neurodegeneration and its clinical manifestations constitute the most pressing challenge, both scientifically and clinically, in developing treatments for MPS. Besides, it is also worth noting that the currently available ERTs have only limited efficacy in treating skeletal, respiratory, and eye lesions [12]. Regarding the long term outcome of MPS, the substance accumulations causing skeletal, respiratory, and cardiac manifestations are known to lead to poor prognosis, probably much more so than other symptoms [13]. Therefore, even if the neurodegeneration as one of the most debilitating aspects of neuropathic MPS can be overcome by novel therapies, the life threatening complications due to the above mentioned lesions remain to be addressed.

These limitations point to the need for further elucidation of neurodegeneration and the subsequent pathogenesis of CNS manifestations on the one hand and for concentrated and multidisciplinary efforts to develop novel therapeutics for neuropathic MPS on the other [14]. Therefore, before reviewing the ongoing efforts to develop innovative therapeutics for neuropathic MPS, this paper first summarizes current understanding of neurodegeneration and its progression towards clinical CNS manifestations. More details regarding the pathophysiology of CNS in MPS are discussed in other papers.

## 3. Central Nervous System Pathology in Neuropathic MPS

### 3.1. Enzyme Deficiency and Substance Accumulation in CNS

Deficiencies in lysosomal enzymes in neuropathic MPS lead to excessive intra- and extra-cellular substance (glucosaminoglycan (GAG)) accumulations, causing widespread tissue and organ dysfunction and systemic structural damage. Four major GAG polysaccharides (heparan sulfate (HS), keratan sulfate (KS), dermatan sulfate (DS), and chondroitin sulfate (CS)) are known to play decisive roles in the pathogenesis of MPS [15,16]. A summary is given below in Table 1 [15,17,18] to illustrate neuropathic MPS, their deficient enzymes, the main deposition of GAG involved, and the corresponding neuropsychiatric and systemic symptom severity.

### 3.2. Neuropathological Progression in Neuropathic MPS

**Figure 1:**
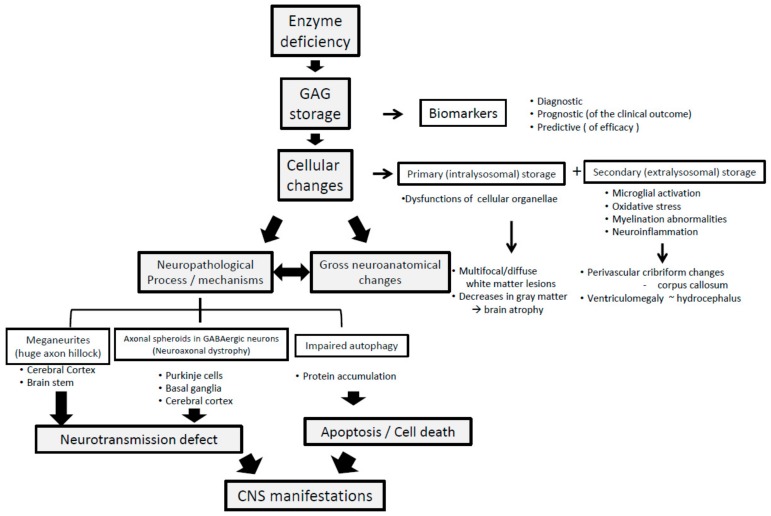
Neuropathological progression in neuropathic MPS.

In contrast to the clear causal relationship between enzyme deficiency and resultant GAG accumulation, the pathogenic processes leading from GAG accumulation to clinical manifestations are complex and multifaceted [15,16,19,20,21]. A schematic presentation of this cascade of events is given in Figure 1, showing how previously reported neuropathological and neurochemical changes progress towards CNS manifestations.

First, GAG accumulation in neurons brings about cellular changes by way of primary (intralysosomal) and secondary (extralysosomal) accumulations, which eventually give rise to gross neuroanatomical changes across various regions in the brain. Additionally, in some types of MPS, e.g., IIB, secondary accumulations of gangliosides contribute further to this process [22]. Probably in parallel, these cellular changes also generate microscopic neuropathological processes, including meganeurites in the cerebral cortex and brain stem and neuroaxonal dystrophy in the GABA (gamma-aminobutyric acid)-ergic neurons, as well as impaired autophagy. The two former events result in neurotransmission defects, whilst impaired autophagy precipitates apoptosis and neuronal death. Furthermore, yet other factors, such as oxidative stress and neuroinflammation, are reported to contribute to neuroanatomical changes [16]. Taken together, the neurotransmission defects and neuronal death, as the two final stages of this neurodegeneration cascade, seem to be mutually responsible for the resulting CNS symptoms. It is not clear, however, how the structural and the functional components interact with each other to cause clinical neuropsychiatric manifestations [23], because neuronal dysfunction might occur independently of the GAG accumulation and structural damage [24]. Generally speaking, the functions and structures of the CNS are closely interconnected, interacting with one another as a network, and cannot be clearly separated [25]. Intuitively, however, the functional component of the CNS is a better target for pharmacotherapy than the structural component, because the latter, including irreversible neuronal loss, is far less amenable to ERT than the former. Therefore, ERT should ideally be initiated as soon as a diagnosis is made so that early neurotransmission defects could be avoided, with lesser likelihood of subsequent neuroanatomical changes. Furthermore, in pediatric patients, CNS manifestations are considered to depend on an intricate balance (or tug of war) between the remaining functional and structural neuroplasticity [26,27] and progressive neurodegeneration. Taken together with their varying levels of disease severity, this implies further difficulty in evaluating drug efficacy and treatment response to ERT in this patient population.

Unlike other neurodegenerative disorders in which lesion localization is specific to each disease (e.g., the nigrostriatal dopaminergic system in Parkinson’s disease [28] and caudate nucleus and putamen in Huntington’s disease [29]), neuropathic MPS is considered to affect the brain in a more global fashion. This is shown in a postmortem report on a patient with MPS-I, which revealed wide substance accumulation throughout both the central and peripheral nervous system and no clear correlation between the neuropathological findings and the clinical picture [30]. This implies that the distribution patterns of GAG accumulation and the resultant neuropathological changes and/or neurotransmission defects may not be disease specific enough to give them pathognomonic or diagnostic value. The CNS manifestations per se do not have substantial diagnostic value either, which may explain why neuropathic MPS is not listed as a distinct neuropsychiatric entity among the congenital metabolic diseases that exhibit neuropsychiatric features [31].

### 3.3. GAG Derived Neuropathology and Its Transition to Clinical CNS Symptoms

Impaired HS catabolism in the CNS, through the above mentioned neuropathological cascade, leads to the formation of a variety of clinical symptoms [15]. CNS manifestations [2,18,19] are markedly diverse, reflecting the complexity of the neuropathological progression, but major ones are optic atrophy, retinopathy, hearing impairment, seizures, and neuropsychiatric symptoms, which include cognitive and behavioral symptoms, neurodevelopmental delays, and sleep disturbances.

Some neuropsychiatric symptoms seem to differ amongst MPS I, II, III, and VII in terms of their personality/behavioral changes [18]. Clinically, these changes, however intricate and fluctuating they may be, can give rise to significant practical difficulties in daily living for the patients, as well as in clinical examinations and treatment procedures, which sometimes require sedation of the patients to control their aggression, impulsivity, and hyperactivity.

## 4. Novel ERTs for Neuropathic MPS

### 4.1. Rationale for Enzyme Delivery across the BBB

Delivering enzymes to the brain to correct enzyme deficiencies is a major therapeutic goal in cases of neuropathic MPS [2]. The main hurdle to overcome in achieving this goal is the BBB. The BBB’s control of vascular permeability is essential in keeping neurotoxic substances and microorganisms out of the CNS whilst allowing necessary compounds in [32], but it is also a significant impediment to delivering therapeutic agents with large molecular weights, such as enzymes, into the brain to treat otherwise incurable CNS diseases. To circumvent the essential physiological functions of the BBB and deliver the enzymes necessary to treat neuropathic MPS, three strategies have been attempted so far.

(1) Enzyme modulation: Enzymes are fused with antibodies for the receptors on the capillary endothelial cells of the brain, allowing endocytosis through the receptors into the endothelial cells, followed by exocytosis into the abluminal space in the brain; this method helps deliver enzymes from the blood within the vascular lumen into the brain parenchyma.

(2) Modified routes of administration: Intrathecal [6,7], intracisternal [33], and intracerebroventricular [8] administrations have been attempted to deliver enzymes directly by circumventing the cerebrovascular circulation involving the BBB.

(3) Dose escalation to increase drug delivery through the BBB: enzymal products are administered at high dosages to maximize the intracerebral concentration and compensate for the loss of some of the administered drug when it is blocked by the BBB.

This paper focuses on the first strategy, enzyme modulation.

### 4.2. Enzyme Modulation by Fusion with Antibodies Enabling Transcytosis

Despite the BBB’s role in blocking external substances from penetrating into the brain parenchyma, some endogenous proteins (e.g., insulin, leptin, and transferrin) cross the BBB through specific receptors expressed on the luminal side of brain capillary endothelial cells [34,35,36]. Therefore, attempts have been made to take advantage of this BBB penetrating system, which is known as receptor mediated transcytosis, to deliver intravenously administered enzymes into the brain parenchyma [37,38].

**Figure 2:**
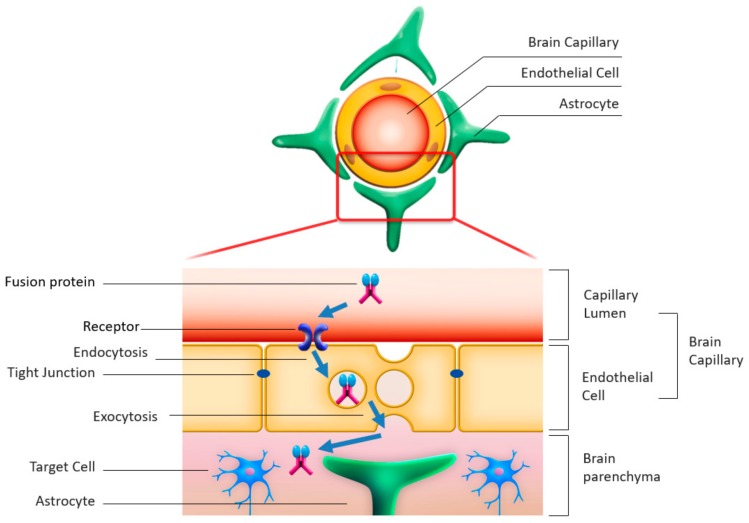
Schematic representation of the transcytosis mechanism.

The putative mechanism of action of transcytosis, by which the delivery of the fusion protein into the brain is achieved, is as follows (Figure 2). First, the fusion protein that consists of the deficient enzyme and the antibody against the endogenous protein receptor binds to the receptor on the luminal side of the brain capillary endothelial cell following its intravenous administration. Second, the fusion protein is incorporated into the endothelial cell by endocytosis. Third, the fusion protein is then exocytosed into the abluminal side of the capillary endothelial cell facing the brain parenchyma, where it detaches from the receptor and exerts its efficacy toward the target cells (neurons).

Monkey and mouse models of MPS-II have respectively provided preclinical evidence of the delivery of enzymes into the brain by transcytosis via insulin receptors [39] and transferrin receptors [40]. While the above cited studies did not reduce GAG accumulations in the CNS, Sonoda et al. used a novel BBB penetrating human iduronate-2-sulfatase (hIDS) called JR-141 to achieve not only distribution in the brains of mice and monkeys, but also reduced GAG levels in the CNS of the model mice [4]. JR-141 and three other compounds have moved on to the clinical developmental phase, as discussed below.

### 4.3. Clinical Trials for Neuropathic MPS

Table 2 summarizes information currently available on seven clinical trials for neuropathic MPS in which various novel approaches to address CNS manifestations have been or will be evaluated. Several reports on the results of these clinical trials have been published [3,5,6,40], yet none of the compounds tested have yet been approved for general clinical use.

There are several notable challenges in the development of novel ERTs for neuropathic MPS. One concerns the issue of the delivery of enzymes across the BBB, as discussed in Section 4.2, and the second, discussed in detail in Section 4.4, concerns methodological issues related to clinical development specific to neuropathic MPS. The third relates to severe non-CNS symptoms, some of which lead to life threatening complications, monitored and recorded in pharmacovigilance operations as serious adverse events unrelated to the test drug. These grave non-CNS symptoms are beyond the reach of ERT, both currently available and in development.

### 4.4. Methodological Challenges in Clinical Development for Neuropathic MPS

(1) Clinicopathological heterogeneity of the patient population:

While neuropathic MPS itself has a fairly homogeneous genetic background, patients with the disease show immense phenotypic heterogeneity in terms of disease progression and severity of clinical CNS manifestations [48]; this heterogeneity is most likely a reflection of the aforementioned neurodegeneration process, which in itself needs further elucidation. Furthermore, the trajectory of the somatic and neurocognitive development of individual children is known to be diverse, against which the natural history of the heterogeneous CNS manifestations in neuropathic MPS [2] needs to be evaluated, as discussed below in detail. In other words, the heterogeneity of the patient population directly leads to theoretical and practical difficulties in assessing disease severity and activity in clinical trials [49].

**Figure 3:**
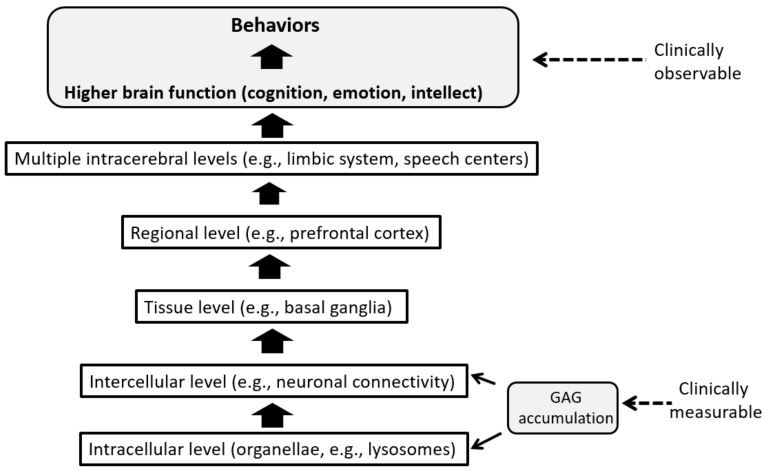
Hierarchical progression of neurodegeneration and CNS manifestations in MPS.

(2) Definition of endpoints and their evaluation:

CNS manifestations, as summarized in Table 2, are currently one of the most difficult aspects of MPS in terms of clinical management. These manifestations represent disorders in higher brain functions that are derived from various pathological conditions at different levels in the CNS. Figure 3 schematically illustrates the hierarchical layers of CNS structures through which initial GAG accumulations induce a series of pathological processes upwards that finally culminate in behavioral abnormalities that are clinically observable.

There is no knowing at the moment which neurological and psychiatric symptoms reflect neuronal dysfunction versus neuronal loss at which level in this hierarchy. As a result, “informed guesswork” [23] based on animal model data guides the choice of clinical endpoints. This process, therefore, seems to be still far from a precise science. One option would be to pick endpoints that reflect fundamental pathophysiology and also correlate to clinically observable behavioral changes resulting from the complex processes of neurodegeneration. In the hierarchical model above, evaluation of the starting point in the downstream (GAG accumulation) and of the ultimate upstream symptom formation (CNS manifestations) will, hopefully, allow us to deduce what takes place between these two ends. Indeed, this has been the common developmental strategy in the aforementioned clinical trials for neuropathic MPS: assessment of the composite endpoints of GAG concentrations in the blood, urine, and CSF on the one hand and of the neurocognitive endpoints on the other [47,49]. Although this seems to be one of the few pragmatically reasonable and theoretically acceptable options, practical difficulties still remain: the neurodevelopmental trajectory requires many years to evaluate, and there is large individual variation in severity and disease progression across the patient population.

Regarding the neurodevelopmental trajectory, a precise evaluation of the neurocognitive effects of new treatments ideally requires a comparison of three different trajectories: (1) the normal neurocognitive development in very young children, (2) the natural history of neurocognitive impairment in the patients with neuropathic MPS, and (3) the observed neurodevelopmental trajectory of the MPS patients receiving a novel ERT in its clinical trial. In practice, this comparison involves a number of further issues, e.g., limited availability of the natural history data [50], different test batteries used in natural history studies and clinical trials across countries, and the time constraint in conducting clinical trials that limits the length of available time to monitor the developmental trajectory. For example, amongst the five published clinical trials of neuropathic MPS, two studies [5,41] did not include neurocognitive assessment, whilst the remaining three trials [3,6,47] slightly differed in their choice of assessment batteries, albeit using one method in common (the Bailey Scales of Infant and Toddler Development, Third Edition). The natural history data [51], on the other hand, utilize three standardized tests to evaluate cognitive functions (The Mullen Scales of Early Learning, The Differential Ability Scale, and The Leiter International Performance Scale) that can provide data comparable and equivalent to some, but not all, data from the Baily Scale used in other studies. Interpretation of the data from these comparable, but different scales across trials may run the risk of both alpha and beta errors, although, by the very nature of rare diseases, efforts need to be made to maximize the value of all the available valuable datasets from the clinical trials on neuropathic MPS, in order to expedite the development of much coveted novel therapies.

One potential solution to enable successful completion of clinical trials leading to a timely regulatory approval for these rare and severe diseases on the one hand and a reasonably robust longitudinal data analysis comparing the developmental trajectories on the other would be a post-approval collection of neurocognitive data from the patients enrolled in the clinical trials. In this way, the pre- and post-approval neurocognitive datasets can be combined to maximize their values for the longitudinal efficacy evaluation of novel ERT for neurocognitive impairment even in the limited patient population with marked heterogeneity.

## 5. Paths Forward

In addition to neuropathic MPS, there are many lysosomal storage disorders (LSDs) with CNS involvement, all of which are in need of effective treatments to deal with the associated neurodegeneration. Therefore, the challenges facing researchers in the development of novel ERTs for neuropathic MPS apply equally to those seeking ways to treat other LSDs involving the CNS. To address the multi-systemic symptoms of LSDs, a new polyvalent therapeutic strategy [21] based on the novel ERT described in this paper is expected to offer significant therapeutic advantages over current ERT, which has no efficacy against CNS symptoms.

To deal with these challenges, multidisciplinary approaches are essential. From the clinical point of view, a wide range of medical specialties needs to be involved in dealing with the variety of clinical manifestations. In particular, to better understand, evaluate, and take care of the CNS symptoms in neuropathic MPS, neurology and psychiatry, neurolinguistics, neuropsychology, and neuroimaging, among other subspecialties, are indispensable. Developing and manufacturing novel enzymes that can cross the BBB to reach the CNS will require advanced knowledge and state-of-the-art technologies from the fields of molecular genetics, immunology, and proteomics and timely responses to new guidelines and regulations applying to rare diseases [52]. International collaboration across multiple organizations ranging from academia, health care providers, industry, governmental regulatory authorities, and patient advocacy groups will also be critical in overcoming the many hurdles that must be surmounted in developing novel treatments for such severe and rare diseases as MPS.

## Figures and Tables

**Table 1:** Biochemical classification of neuropathic mucopolysaccharidoses (MPS) and corresponding symptom severity. GAG, glucosaminoglycan; HS, heparan sulfate; DS, dermatan sulfate.

Neuropathic MPS	Deficient Enzyme	Main GAG Stored	Neuropsychiatric Symptoms	Systemic Symptoms
MPS-I (Hurler syndrome; Hurler–Scheie syndrome; Scheie syndrome)	α-L-iduronidase (IDUA)	HS, DS	Hurler: severe Hurler–Scheie and Scheie: mild to absent	Wide spectrum of severity
MPS-II (Hunter syndrome)	Iduronate-2-sulfatase (IDS)	HS, DS	Severe (rapidly progressing phenotypes) or mild to absent (slowly progressing phenotypes)	Wide spectrum of severity
MPS-IIIA (Sanfilippo A syndrome)	Heparan-N-sulfatase (SGSH)	HS	Severe	Mild to absent
MPS-IIIB (Sanfilippo B syndrome)	α-N-acetylglucosaminidase (NAGLU)	HS	Severe	Mild to absent
MPS-IIIC (Sanfilippo C syndrome)	α-glucosaminidase acetyltransferase (HGSNAT)	HS	Severe	Mild to absent
MPS-IIID (Sanfilippo D syndrome)	N-acetylglucosamine 6-sulfatase (GNS)	HS	Severe	Mild to absent
MPS-VII	β-D-glucuronidase (GUSB)	HS, DS	Severe (rapidly progressing phenotypes) or mild to absent (slowly progressing phenotypes)	Wide spectrum of severity

**Table 2:** Hitherto attempted enzyme replacement therapies targeting CNS pathology in MPS. ERT, enzyme replacement therapy.

MPS	Compound	Targeted Receptor to Cross BBB	Route of Administration	Developmental Status	Manufacturer/Sponsor	Source
MPS-I (Hurler syndrome)	Valanafusp alpha	Insulin receptor	Intravenous	Ph I/II trial completed	ArmaGen	Giugliani et al. [3]
	Iduronidase	N/A	Intrathecal (iv ERT + HCT + intrathecal ERT)	An open label Ph I trial	Sanofi Genzyme/NIH	Eisengart et al. [6]
MPS-II (Hunter syndrome)	Idursulfase-IT	N/A	Intrathecal	Ph I/II trial completed	Shire	Muenzer et al. [41]
	DNL 310	Transferrin receptor	Intravenous	Ph I/II trial planned	Denali therapeutics	Press release [42]
	Idursulfase- beta ICV	N/A	Intracerebroventricular	Ph I/II trial completed	Greencross	Press release [43]
	AGT-182	Insulin receptor	Intravenous	Ph I trial completed	ArmaGen	NCT02262338 [44]
	JR-141	Transferrin receptor	Intravenous	Ph I/II completed Ph III ongoing	JCR pharmaceuticals	Okuyama et al. [5]
MPS-IIIA (Sanfilippo A syndrome)	SOBI003	N/A	Intravenous	Ph I/II ongoing	Swedish Orphan Biovitrum (Sobi)	NCT03811028 [45]
MPS-IIIB (Sanfilippo B syndrome)	Tralesinidase alfa (BMN 250)	N/A	Intracerebroventricular	Ph I/II ongoing	BioMarin	NCT02754076 [46]
	SBC-103	N/A	Intravenous	Ph I/II completed	Alexion	Whitley et al. [47]
